# Physical activity as a modest behavioral pathway between parental marital conflict and adolescent anxiety: exploring gender differences

**DOI:** 10.3389/fpsyg.2026.1742433

**Published:** 2026-08-31

**Authors:** Zheng Zhang, Jing Wang, Yongxian Jing, Wanqin Zang, Wan Ahmad Munsif Wan Pa

**Affiliations:** 1 College of Sports Science, Nantong University, Nantong, Jiangsu, China; 2 Faculty of Education, Universiti Kebangsaan Malaysia, Bangi, Selangor, Malaysia; 3 Department of Anesthesiology for Surgery, Xilingol League Central Hospital, Xilinhot, Inner Mongolia, China; 4 Department of Urology, Xilingol League Central Hospital, Xilinhot, Inner Mongolia, China; 5 School of Sports Science, Jishou University, Jishou, China

**Keywords:** adolescents, anxiety, gender, parental marital conflict, physical activity

## Abstract

**Background and objective:**

Parental marital conflict has been consistently associated with adolescent anxiety, but the behavioral correlates that may help explain this association remain insufficiently understood. This study re-examined the association between parental marital conflict and adolescent anxiety and further tested whether physical activity served as a modest statistical mediator and whether gender moderated the association between parental marital conflict and physical activity.

**Methods:**

A cross-sectional self-reported survey was conducted with 2,145 adolescents in China. The survey assessed parental marital conflict, anxiety symptoms, physical activity, and demographic characteristics. Descriptive statistics, correlation analyses, mediation analysis, and moderated mediation analysis were performed using SPSS 26.0 and Hayes’ PROCESS macro with 5,000 bootstrap samples. Age, grade, place of residence, only-child status, and boarding status were included as covariates.

**Results:**

Parental marital conflict was positively correlated with adolescent anxiety (*r* = 0.293, *p* < 0.001) and negatively correlated with physical activity (*r* = −0.072, *p* < 0.001), while physical activity was negatively correlated with anxiety (*r* = −0.107, *p* < 0.001). In the mediation model, parental marital conflict was negatively associated with physical activity (*β* = −0.070, SE = 0.021, *p* = 0.001), and physical activity was negatively associated with anxiety (*β* = −0.083, SE = 0.021, *p* < 0.001). The indirect effect was statistically significant but small [effect = 0.006, BootSE = 0.002, 95% CI (0.002, 0.011)], accounting for approximately 2.06% of the total effect. Gender moderated the association between parental marital conflict and physical activity (*β* = 0.073, SE = 0.021, *p* < 0.001).

**Conclusion:**

The findings suggest that physical activity may represent a modest behavioral correlate in the association between parental marital conflict and adolescent anxiety, and that this pathway may differ by gender. Given the cross-sectional design and the small indirect effect, the results should be interpreted cautiously and should not be taken as evidence of causal protection.

## 1 Introduction

The Global Burden of Disease Study identifies anxiety as a widespread mental disorder and a major contributor to disability (Baxter et al., 2014; Kessler et al., 2005). It commonly emerges during adolescence (Davies et al., 2022), yet many individuals fail to receive adequate treatment (Merikangas et al., 2011), making it a global public health concern (Kalin, 2021). Evidence indicates that the prevalence of anxiety among adolescents is 31.9% in the United States and 37.4% in China (Merikangas et al., 2010; Zhou et al., 2020). The lifetime rate is approximately 32%, with 8.3% experiencing severe psychological impairment (Merikangas et al., 2010). Moreover, anxiety rates continue to increase (Merikangas et al., 2009). Typical symptoms include excessive worry, attention difficulties, and social avoidance (Narmandakh et al., 2021), which are associated with higher risks of substance dependence and suicidal behavior (Alonso et al., 2018). Hence, examining factors such as physical activity and parental marital conflict is essential for understanding and addressing adolescent anxiety.

Previous studies have consistently shown that parental marital conflict is associated with negative emotional outcomes in adolescents, particularly anxiety (Pan et al., 2026; Yan et al., 2026a; Yang and Liu, 2026). Parental marital conflict refers to disagreements or disputes arising from various issues in family life, including both physical and emotional conflicts, and is widely regarded as an important indicator of family functioning and quality of family life (Cummings and Davies, 2002). According to family systems theory, the family operates as an interconnected emotional unit, such that conflict between parents may disrupt the emotional balance of the family system and affect children’s psychological adjustment (Bowen, 1966). When adolescents perceive instability or tension in the family environment, they may be more likely to experience negative emotions, including anxiety and depression (Yang et al., 2016). Empirical studies have further confirmed that parental marital conflict is associated with an increased risk of adolescent anxiety, and longitudinal evidence suggests that it may also predict anxiety over time (Bi et al., 2017; Grover et al., 2005; Spence et al., 2002). Accordingly, this direct association should be regarded as relatively well established rather than as the sole novelty of the present study. The current study therefore re-examined this association in the present adolescent sample while extending prior research by testing whether physical activity functions as a modest behavioral pathway and whether gender conditions this pathway.

Physical activity refers to voluntary bodily movement produced by skeletal muscles that results in energy expenditure, including both structured exercise and unstructured movement in daily life (Wang et al., 2025a). In the present study, physical activity was conceptualized as adolescents’ overall activity level rather than participation in any single specific exercise type, and it was assessed across three dimensions: intensity, duration, and frequency. However, persistent physical inactivity among children and adolescents has become a global public health concern (Aubert et al., 2021). Parents are an important socializing influence on adolescent physical activity. Meta-analytic evidence indicates that parents shape adolescents’ activity levels through encouragement, role modeling, emotional support, and the provision of exercise opportunities and resources (Su et al., 2022). When parental relationships are characterized by high levels of conflict, family functioning may be disrupted, particularly in terms of parent–child communication, emotional responsiveness, supervision, and involvement in shared activities, thereby reducing adolescents’ opportunities and motivation to engage in physical activity (Liang and Chen, 2025). This pathway can be understood from several complementary theoretical perspectives. Emotional Security Theory suggests that chronic interparental conflict may undermine adolescents’ sense of safety and emotional security within the family. Self-Determination Theory further suggests that high-conflict family environments may weaken the satisfaction of adolescents’ basic psychological needs for autonomy, competence, and relatedness, thereby reducing intrinsic motivation for physical activity (Ryan et al., 2022). In addition, Social Learning Theory posits that adolescents may acquire maladaptive coping patterns, such as avoidance or withdrawal, through observing parents’ responses to chronic conflict, which may further discourage active behavioral engagement (Mobley and Sandoval, 2008). Taken together, these perspectives suggest that parental marital conflict may create a family context that is less supportive of physical activity and more conducive to inactivity or withdrawal.

A substantial body of evidence has shown that physical activity is associated with better physical and mental health outcomes (Gan et al., 2025; Liu et al., 2024c; Luo et al., 2025; Peng et al., 2025a; Yan et al., 2026b; Yang et al., 2025a; Yi et al., 2025; Yin et al., 2025). In particular, anxiety, a common mental health problem in adolescence, has been found to be closely associated with physical activity levels (Liu et al., 2024b,d, 2025; Wang et al., 2025b; Xiao et al., 2024). Adolescents with higher levels of physical activity tend to report lower anxiety, whereas insufficient physical activity has been identified as a risk factor for anxiety symptoms and other mental health problems such as depression (Jia et al., 2025; Liu et al., 2024a; Peng et al., 2025b,c; Shen et al., 2024; Wang W. et al., 2025; Yang et al., 2025b). Physical activity may be associated with lower anxiety through several pathways, including enhanced self-efficacy, improved sleep quality, better emotion regulation, and stronger social connectedness. Systematic reviews have likewise supported the role of physical activity in reducing anxiety symptoms (Moreno-Peral et al., 2022). At the same time, some studies suggest that adolescents with higher anxiety may be less likely to engage in physical activity because of avoidance, low motivation, and social withdrawal (DeWolfe et al., 2023). Although this indicates that the association may be bidirectional, the present study focuses on whether physical activity may serve as one potential behavioral pathway linking parental marital conflict to adolescent anxiety. Taken together, theory and prior empirical findings suggest that parental marital conflict may be associated with lower physical activity by undermining adolescents’ emotional security, weakening family-based motivational support, and modeling maladaptive coping patterns, while lower physical activity may in turn be associated with higher anxiety. On this basis, the present study hypothesized that physical activity would mediate the association between parental marital conflict and adolescent anxiety.

According to Gender Role Theory, individuals acquire gender-related behavioral norms, emotional expression styles, and stress-coping strategies through socialization processes (Eagly and Wood, 2016). This perspective suggests that gender may shape not only how adolescents perceive and respond to familial conflict but also the coping strategies they are more likely to use under stress, including the extent to which they engage in physical activity (Kim, 2021; Wang et al., 2020). Male adolescents are generally more likely to adopt active or externalizing coping styles, whereas female adolescents are more likely to rely on internalizing strategies such as emotional suppression, self-reflection, and seeking emotional support (Yoon et al., 2023). Because physical activity is more common among boys and is also more often perceived as a means of emotional release, boys may be more behaviorally sensitive to family stress when this coping pathway is disrupted (Kretschmer et al., 2023). Under conditions of frequent or intense parental conflict, emotional distress may reduce their motivation to engage in previously preferred coping behaviors, including physical activity (Jiang et al., 2021). By contrast, female adolescents may be less affected in this pathway because their coping strategies tend to rely less directly on physical activity. Based on this theoretical and empirical foundation, the present study hypothesizes that gender moderates the relationship between parental marital conflict and adolescent physical activity.

**Figure 1:**
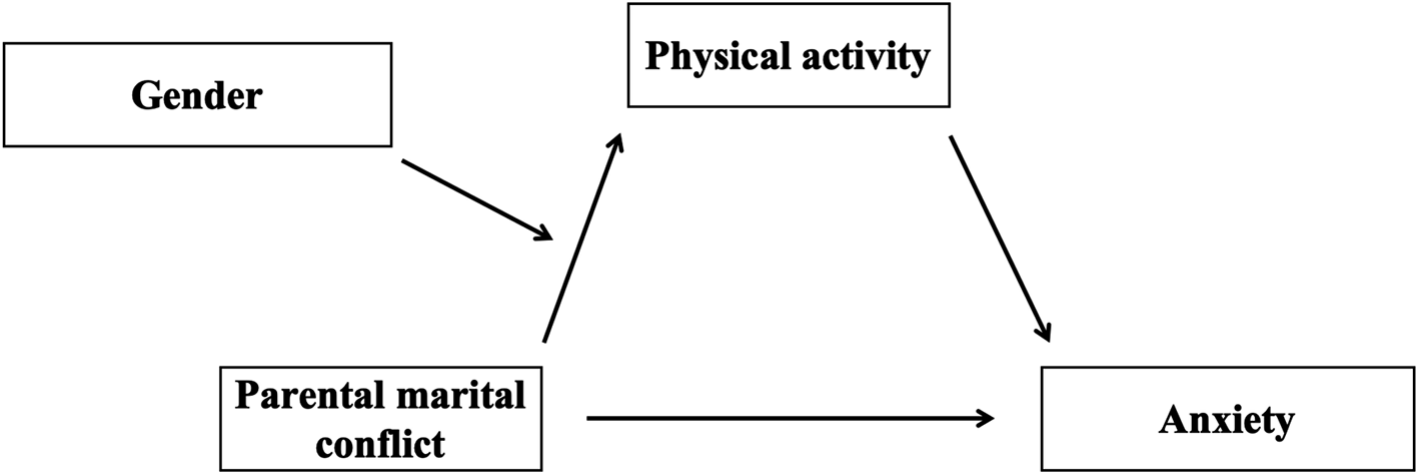
Hypothesized a mediation model.

While existing research has explored the association between parental marital conflict and adolescent anxiety, the specific pathway involving physical activity and the potential gender difference in this pathway remain underexplored. To address this limitation, the current study developed a moderated mediation model, in which physical activity was tested as a modest statistical mediator and gender was specified as a moderator of the path from parental marital conflict to physical activity. This model was selected because gender differences in adolescent physical activity are well documented and because the theoretical rationale is strongest for gender differences in behavioral coping responses to family stress, rather than for gender differences in the association between anxiety symptoms and physical activity. To avoid overstatement, physical activity is conceptualized here as a potentially modifiable behavioral correlate rather than as a strong causal protective mechanism (see Figure 1).

## 2 Methods

### 2.1 Participants

A cross-sectional survey was conducted from September to October 2024 using a convenience sampling method. The study targeted adolescents from South China, Southwest China, and Central China, and a total of 2,309 participants were initially recruited. Data were collected through paper-based questionnaires administered in classroom settings. Prior to participation, the purpose of the study, the anonymous and confidential nature of the data, and its intended use were clearly explained. Informed consent was obtained from all participants and their legal guardians.

The average time required to complete the questionnaire was approximately 10 min. Ethical approval was obtained from the Medical Ethics Committee of the authors’ affiliated institution before data collection commenced, ensuring full compliance with ethical and legal standards. All research procedures adhered to the institutional ethical guidelines, thereby enhancing the study’s reliability and participants’ trust.

During data processing, all returned questionnaires were rigorously screened for quality. Responses exhibiting patterns of mechanical answering (e.g., repeated selection of the same option or wave-shaped response patterns), or containing substantial missing data, were excluded. After screening, a total of 2,145 valid questionnaires were retained for analysis. Among the respondents, 1,038 were male and 1,107 were female, with a mean age of 14.83 years (SD = 1.313) (see Table 1).

**Table 1:** Baseline characteristics of participants by gender.

Characteristic	Overall (*N* = 2,145)	Boys (*n* = 1,038)	Girls (*n* = 1,107)	*p* value
Age, M (SD)	14.83 (1.31)	14.68 (1.31)	14.98 (1.30)	< 0.001
Grade				< 0.001
Primary school	105 (4.9%)	71 (6.8%)	34 (3.1%)	
Junior high school	842 (39.3%)	474 (45.7%)	368 (33.2%)	
Senior high school	1,198 (55.9%)	493 (47.5%)	705 (63.7%)	
Place of residence				0.020
Towns	763 (35.6%)	343 (33.0%)	420 (37.9%)	
Village	1,382 (64.4%)	695 (67.0%)	687 (62.1%)	
Only-child status				0.534
Yes	147 (6.9%)	67 (6.5%)	80 (7.2%)	
No	1,998 (93.1%)	971 (93.5%)	1,027 (92.8%)	
Live on campus				0.368
Yes	1,768 (82.4%)	864 (83.2%)	904 (81.7%)	
No	377 (17.6%)	174 (16.8%)	203 (18.3%)	

*p* values were obtained from an independent-samples *t* test for age and chi-square tests for categorical variables.

### 2.2 Measures

#### 2.2.1 Parental marital conflict

Parental marital conflict was measured using the Children’s Perception of Interparental Conflict Scale (CPIC), originally developed by Grych et al. (1992) and later revised for Chinese populations by Chi and Xin (2003). The revised version contains five items that assess adolescents’ perceptions of conflict between their parents. Each item is rated on a 4-point Likert scale ranging from 1 (strongly disagree) to 4 (strongly agree). Total scores range from 5 to 20, with higher scores indicating greater perceived parental marital conflict. In the present study, the scale demonstrated good internal consistency, with a Cronbach’s *α* coefficient of 0.779.

#### 2.2.2 Anxiety

Anxiety symptoms over the past 2 weeks were assessed using the Generalized Anxiety Disorder Scale-2 (GAD-2). The GAD-2 includes two core items assessing the frequency of feeling nervous, anxious, or on edge and being unable to stop or control worrying. Each item is rated on a 4-point Likert scale ranging from 1 (not at all) to 4 (nearly every day), yielding a total score between 2 and 8, with higher scores indicating more severe anxiety symptoms (Byrd-Bredbenner et al., 2021; Kroenke et al., 2007; Plummer et al., 2016; Skapinakis, 2007). The GAD-2 was selected because it captures core anxiety symptoms while substantially reducing respondent burden and has shown acceptable screening performance in previous psychometric and meta-analytic studies. This feature is particularly appropriate for the present school-based adolescent sample, which included primary, junior high, and senior high school students, because questionnaires had to be completed within limited classroom time and excessive survey length may increase fatigue or careless responding. Given the considerable academic demands faced by Chinese adolescents, a concise screening measure was considered suitable for improving the feasibility and quality of data collection. In this study, the GAD-2 was used to assess anxiety symptom severity rather than to establish a clinical diagnosis. The internal consistency for this sample was high, with a Cronbach’s *α* of 0.810.

#### 2.2.3 Physical activity

Physical activity was assessed using the Physical Activity Questionnaire developed by Liang (1994), which has been widely used in previous research involving Chinese adolescents (Peng et al., 2025c; Wang W. et al., 2025). In the present study, physical activity was conceptualized as an overall indicator of adolescents’ activity level rather than as separate categories of light-, moderate-, or vigorous-intensity physical activity. The questionnaire assesses three dimensions of physical activity: intensity, duration, and frequency. The intensity item includes five response options representing progressively higher levels of activity intensity, with representative examples provided in the original scale, while the other two items assess the usual duration of each activity session and the frequency of participation. Intensity and frequency were rated on 5-point scales ranging from 1 to 5, while duration was rated on a 5-point scale ranging from 0 to 4. A composite physical activity score was calculated by multiplying the scores of these three dimensions, with higher scores indicating higher overall levels of physical activity. This measure was selected because it captures multiple behavioral aspects of physical activity commonly considered in adolescent research, while also using a relatively brief format that helps reduce respondent burden in large-scale survey settings. In the current study, the scale showed modest internal consistency, with a Cronbach’s *α* of 0.608. Although this value is not high, it is still within an acceptable range for a brief measure with fewer than six observed items (Hair, 2009). Previous methodological literature has suggested that, in such cases, a Cronbach’s α coefficient above 0.60 may indicate acceptable reliability. At the same time, this relatively modest internal consistency may partly reflect the structure of the measure itself. Specifically, intensity, duration, and frequency represent related but distinct behavioral aspects of physical activity rather than highly similar or interchangeable items. Because these components capture different dimensions of adolescents’ activity patterns, their inter-item similarity may be limited, which may in turn result in a relatively lower Cronbach’s α (Raykov, 2012; Sijtsma, 2009).

### 2.3 Data processing and analysis

All statistical analyses were conducted using SPSS version 26.0. First, common method bias was assessed. A threshold below 40% was used to indicate the absence of significant common method variance (Podsakoff et al., 2003). Descriptive statistics and Pearson correlation analyses were performed for demographic variables and primary study constructs. Because gender was examined as a moderator, baseline characteristics were reported separately for boys and girls. Independent-samples t tests or chi-square tests were used for preliminary group comparisons, and exact *p* values were reported whenever possible. Prior to hypothesis testing, all continuous variables were standardized. To examine the hypothesized relationships, Hayes’ PROCESS macro in SPSS (Models 4 and 7) was employed to test the mediating role of physical activity and the moderating role of gender (Hayes, 2018). Age, grade, place of residence, only-child status, and boarding status were included as covariates because these demographic and family/school-context characteristics may be associated with adolescents’ family experiences, physical activity opportunities, and anxiety symptoms. The model was estimated using 5,000 bootstrap resamples to calculate bias-corrected 95% confidence intervals (95% CI), ensuring robustness of the results (Berkovits et al., 2000). Because the physical activity score was calculated by multiplying intensity, duration, and frequency, its distribution was additionally examined for skewness and extreme values. Sensitivity analyses using log-transformed physical activity scores and winsorized physical activity scores were conducted to assess whether the mediation findings were robust to distributional non-normality. A significance level of 0.05 was used throughout the analyses.

## 3 Results

### 3.1 Common method bias test

The results of the common method bias test revealed three factors with eigenvalues greater than 1. The first factor accounted for 26.93% of the total variance, which is below the 40% threshold. This suggests that the study does not face significant common method bias risk.

### 3.2 Descriptive analysis

As shown in Table 2, demographic variables were included to describe sample characteristics and to provide preliminary group comparisons for parental marital conflict, anxiety, and physical activity. Exact *p* values were added to improve transparency. A significant difference in parental marital conflict was found by place of residence (*t* = 3.07, *p* = 0.002), with adolescents living in urban areas reporting higher levels of parental marital conflict than those living in rural areas. A significant gender difference was also found in physical activity (*t* = 9.34, *p* < 0.001), with boys reporting higher levels of physical activity than girls. No significant differences were observed for anxiety across gender, residence, only-child status, or boarding status. The physical activity score showed a right-skewed distribution (*M* = 15.67, SD = 18.42, median = 10.00, IQR = 4.00–20.00, skewness = 2.31, excess kurtosis = 6.40), with values ranging from 0 to 100. Using the conventional 1.5 × IQR rule, 166 observations were above the upper fence of 44. Sensitivity analyses showed that the indirect effect remained statistically significant when physical activity was log-transformed [effect = 0.007, BootSE = 0.003, 95% CI (0.002, 0.012)] or winsorized at the upper-fence value [effect = 0.007, BootSE = 0.003, 95% CI (0.003, 0.013)]. Thus, the direction and significance of the mediation result were robust, although the indirect effect remained small.

**Table 2:** Group comparisons of main study variables with exact *p* values.

Group/statistic	Category	Parental marital conflict	Anxiety	Physical activity
Gender	Boys	10.79 (3.53)	3.87 (1.72)	19.43 (21.51)
	Girls	10.75 (3.48)	3.94 (1.69)	12.15 (14.09)
	*t*	0.27	−0.96	9.34
	*p*	0.784	0.339	< 0.001
Place of residence	Towns	11.09 (3.40)	3.94 (1.74)	16.05 (19.68)
	Village	10.60 (3.54)	3.89 (1.69)	15.47 (17.70)
	*t*	3.07	0.66	0.70
	*p*	0.002	0.510	0.486
Only-child status	Only children	10.27 (3.41)	3.65 (1.55)	17.78 (21.62)
	Non-only children	10.81 (3.51)	3.92 (1.72)	15.52 (18.16)
	*t*	−1.80	−1.90	1.44
	*p*	0.072	0.057	0.150
Boarding status	Live on campus	10.73 (3.50)	3.93 (1.72)	15.75 (17.95)
	Not live on campus	10.96 (3.50)	3.81 (1.64)	15.31 (20.51)
	*t*	−1.12	1.23	0.42
	*p*	0.261	0.219	0.674

Values are presented as M (SD). Exact *p* values are reported to three decimal places, except for *p* < 0.001.

### 3.3 Correlation analysis

The results presented in Table 3 show that parental marital conflict was positively correlated with adolescent anxiety (*r* = 0.293, *p* < 0.001) and negatively correlated with physical activity (*r* = −0.072, *p* < 0.001). In addition, adolescent anxiety was negatively correlated with physical activity (*r* = −0.107, *p* < 0.001).

**Table 3:** Pearson correlation analysis of parental marital conflict, anxiety, and physical activity.

Variables	1	2	3
1 Parental marital conflict	–	–	
2 Anxiety	0.293***	–	–
3 Physical activity	−0.072***	−0.107***	–

***p < 0.001.

### 3.4 Mediation model test

**Figure 2:**
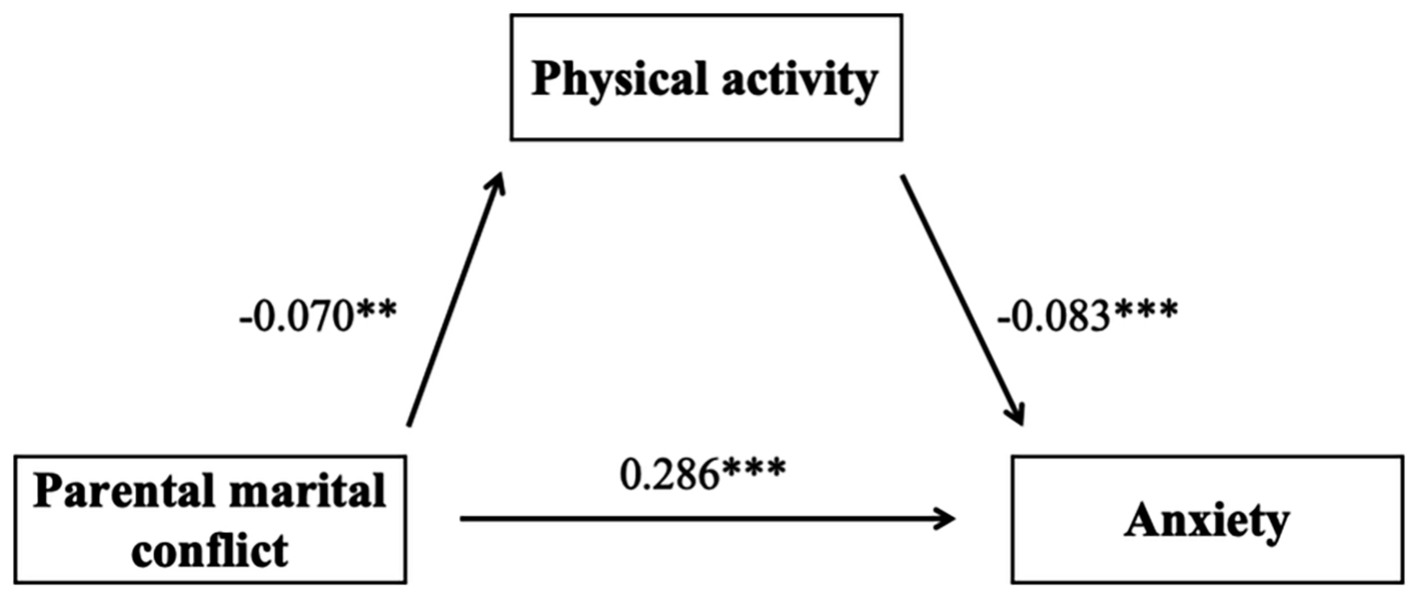
Mediation model of physical activity in the association between parental marital conflict and adolescent anxiety. Standardized path coefficients are presented. All displayed paths were statistically significant (**p < 0.01; ***p < 0.001).

After controlling for age, grade, place of residence, only-child status, and boarding status, the results in Table 4 show that parental marital conflict was significantly positively related to adolescent anxiety (*β* = 0.291, *p* < 0.001). After introducing physical activity as a mediating variable, parental marital conflict remained significantly positively associated with adolescent anxiety (*β* = 0.286, *p* < 0.001). Furthermore, parental marital conflict was significantly negatively related to adolescent physical activity (*β* = −0.070, *p* = 0.001), and physical activity was significantly negatively related to adolescent anxiety (*β* = −0.083, *p* < 0.001). The specific mediation path proportions are shown in Table 5, and the detailed effect model paths are presented in Figure 2. The indirect effect through physical activity was statistically significant but very small, accounting for approximately 2.06% of the total effect.

**Table 4:** Mediation model test.

Outcome variable	Predictor variable	*β*	SE	*t*	*R* ^2^	*F*
Physical activity	Parental marital conflict	−0.070	0.021	−3.241**	0.022	8.054***
Anxiety	Parental marital conflict	0.291	0.021	14.072***	0.090	35.392***
Anxiety	Parental marital conflict	0.286	0.021	13.808***	0.097	32.797***
	Physical activity	−0.083	0.021	−3.969***		

Covariates included age, grade, place of residence, only-child status, and boarding status. **p < 0.01; ***p < 0.001.

**Table 5:** Mediation model path analysis.

Intermediate path	Effect size	SE	Bootstrap 95% CI	Proportion of total effect
Total effect	0.291	0.021	0.251, 0.332	
Total direct effect	0.286	0.021	0.245, 0.326	97.94%
Total indirect effect	0.006	0.002	0.002, 0.011	2.06%

Covariates included age, grade, place of residence, only-child status, and boarding status.

### 3.5 Moderated mediation test

**Figure 3:**
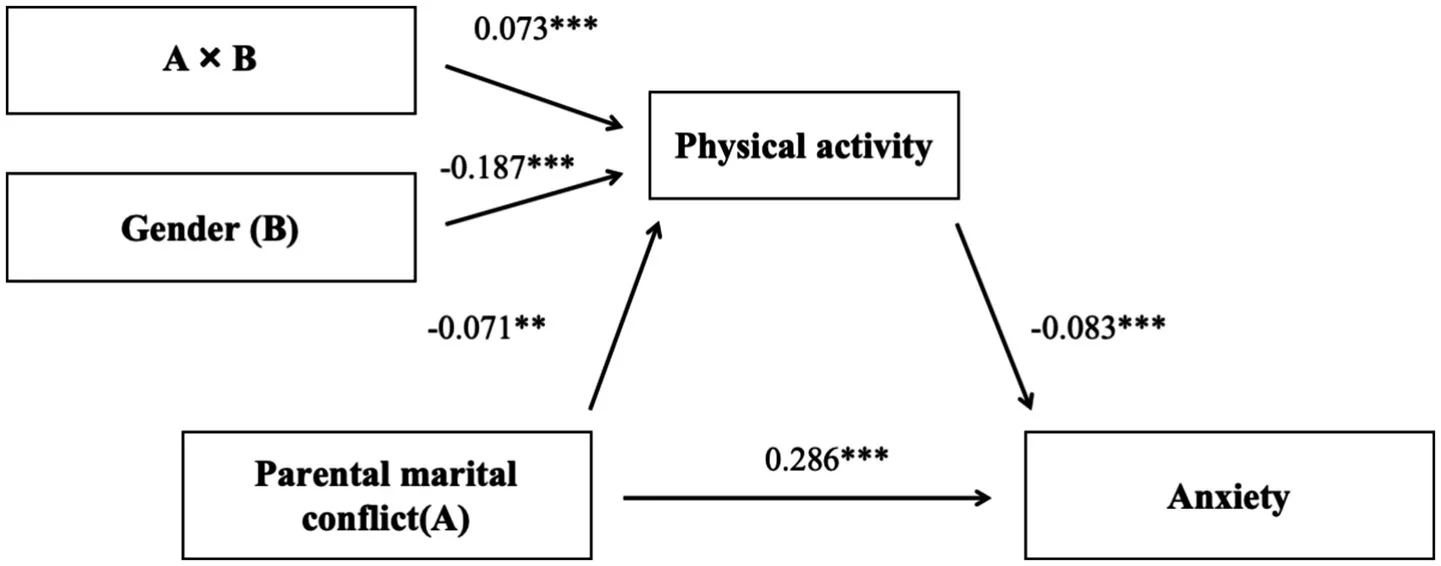
Moderated mediation model of parental marital conflict, physical activity, anxiety, and gender (*p<0.05; **p < 0.01; ***p < 0.001).

**Figure 4:**
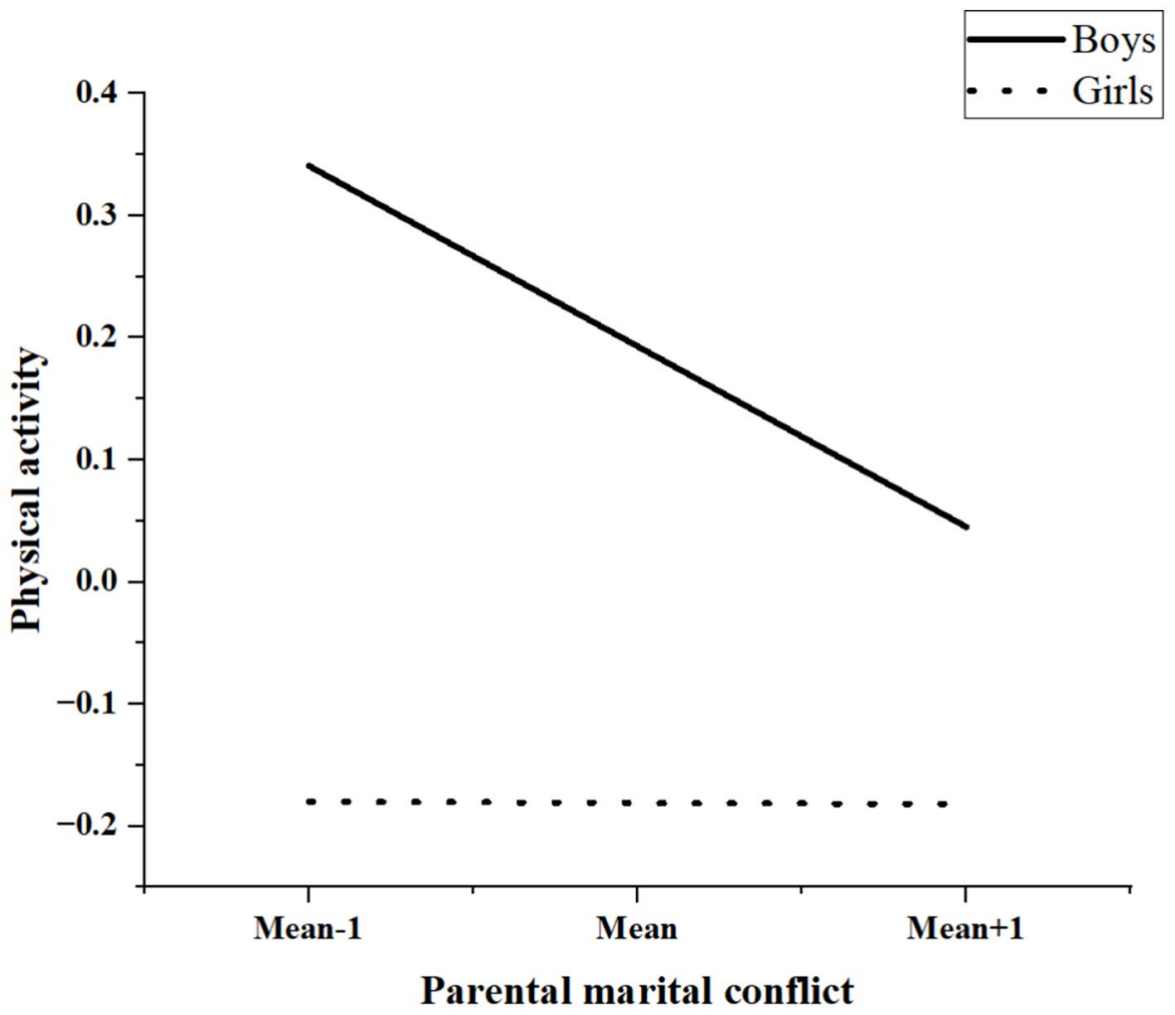
Simple slope analysis of the moderating effect of gender on the association between parental marital conflict and physical activity.

The results presented in Table 6 and Figures 3, 4 show that after including the moderating variable and controlling for age, grade, place of residence, only-child status, and boarding status, parental marital conflict remained significantly negatively related to adolescent physical activity (*β* = −0.071, *p* < 0.001). Gender was also significantly negatively related to adolescent physical activity (*β* = −0.187, *p* < 0.001), and the interaction term between gender and parental marital conflict was significantly related to adolescent physical activity (*β* = 0.073, *p* < 0.001). Further analysis revealed that gender moderated the relationship between parental marital conflict and adolescent physical activity. Simple slope analysis indicated that the negative association between parental marital conflict and physical activity was significant among boys (*β* = −0.147, SE = 0.030, *p* < 0.001) but not among girls. As a sensitivity check for the model specification, additional interaction terms testing whether gender moderated the physical activity-anxiety path or the direct parental marital conflict-anxiety path were not statistically significant (physical activity × gender: *β* = −0.014, *p* = 0.535; parental marital conflict × gender: *β* = 0.036, *p* = 0.084). Therefore, the final model retained gender as a moderator of the parental marital conflict-physical activity path only.

**Table 6:** Tests of the moderated mediation model.

Variables	Physical activity			Anxiety		
	*β*	SE	*t*	*β*	SE	*t*
Parental marital conflict (A)	−0.071	0.021	−3.387***	0.286	0.021	13.808***
Gender (B)	−0.187	0.021	−8.810***			
Physical activity				−0.083	0.021	−3.969***
A × B	0.073	0.021	3.494***			
*R* ^2^	0.061			0.097		
*F*	17.455***			32.797***		

Covariates included age, grade, place of residence, only-child status, and boarding status. ***p < 0.001.

Figure 3 presents the moderated mediation model. Gender moderated the association between parental marital conflict and physical activity through the interaction term, indicating that the strength of this association differed between boys and girls. The remaining paths in the model were statistically significant. Figure 4 shows the simple slope analysis for the moderating effect of gender on the association between parental marital conflict and physical activity. As parental marital conflict increased, physical activity decreased more markedly among boys, whereas the decline was weaker and statistically non-significant among girls.

## 4 Discussion

This study found that parental marital conflict was directly associated with adolescent anxiety and was also indirectly associated with anxiety through physical activity. In addition, gender moderated the association between parental marital conflict and physical activity, suggesting differences in this behavioral pathway across male and female adolescents. Given the cross-sectional design and the small magnitude of the indirect effect, these findings should be interpreted as evidence of statistical associations rather than causal pathways.

Emotional Security Theory (EST) provides an important framework for understanding the association between parental marital conflict and children’s and adolescents’ adjustment, and a substantial body of empirical research supports this perspective (Davies and Martin, 2013). According to EST, exposure to parental marital conflict may undermine adolescents’ emotional security within the family system, thereby reducing an important psychological resource for emotion and behavior regulation (Gao, 2023). In this context, adolescents may be more likely to experience negative emotional and behavioral responses, such as anxiety, fear, avoidance, and social withdrawal, which may in turn increase the risk of maladjustment and internalizing problems. Further research suggests that parental marital conflict may also be associated with adolescent mental health through poorer family functioning and lower-quality parent–child relationships (Dong et al., 2022; Pu and Rodriguez, 2021). From an emotional appraisal perspective, adolescents who repeatedly witness intense disputes between their parents or who grow up in high-conflict family environments may be more likely to experience negative emotional and behavioral responses, such as fear, anxiety, withdrawal, and social alienation (Silva et al., 2020). The severity of these responses appears to be closely related to the frequency and duration of prior conflicts. Existing evidence also suggests that adolescents exposed to high-conflict family environments over extended periods may report higher levels of chronic anxiety symptoms (Guassi and Telzer, 2018). In particular, individuals raised in family environments characterized by frequent conflicts, intense hostility, and a lack of effective conflict resolution mechanisms tend to show higher levels of internalizing problems, which may become more evident during adolescence (O'Hara et al., 2023). In summary, parental marital conflict, as an important family risk factor, may be closely associated with adolescent anxiety through multiple interrelated pathways, including lower emotional security, poorer parent–child relationships, and suboptimal family functioning.

This study supports the hypothesis that physical activity statistically mediates the association between parental marital conflict and adolescent anxiety, which is consistent with previous research (Zheng et al., 2020). However, this mediation effect was very small, and physical activity accounted for only approximately 2.06% of the total effect. Parents involved in marital conflict may show lower emotional engagement, greater rejection, harsher discipline, and more social withdrawal, all of which may be associated with lower levels of physical activity among adolescents (D'Onofrio and Emery, 2019). Adolescents exposed to high-conflict family environments may also be more likely to exhibit avoidance and withdrawal, which may be associated with fewer outdoor activities and lower social participation (Song et al., 2023). From a theoretical perspective, physical activity may function as a mediator because parental marital conflict may reduce adolescents’ motivational and psychological resources for engaging in health-promoting behaviors. At the same time, the present findings suggest that this pathway should be understood as a modest behavioral correlate rather than as a central explanatory mechanism. Adolescents’ physical activity is influenced by many factors beyond family conflict, including school-based physical education, academic schedules, peer contexts, neighborhood resources, and individual preferences. These factors may help explain why the indirect effect was statistically significant but limited in magnitude.

Prior studies have also shown that physical activity is associated with lower levels of depression and anxiety (McDowell et al., 2019). This association may be explained by several pathways, including differences in neurophysiological functioning, higher self-efficacy and emotion regulation, better sleep quality, and stronger social connection (Li et al., 2024; Sato et al., 2007; Zhao et al., 2025). The exercise-self-esteem model further suggests that physical activity may be linked to lower negative emotional experiences through higher self-esteem (Wong et al., 2021). For adolescents, the sense of accomplishment, belonging, and social interaction gained through exercise may be particularly relevant to emotional adjustment. Existing studies likewise indicate that exercise interventions are associated with multiple aspects of adolescent mental health, including emotional stability, self-perception, and environmental adaptation (Ruiz-Ranz and Asín-Izquierdo, 2025). Taken together, these findings suggest that physical activity represents a very modest behavioral pathway in the association between parental marital conflict and adolescent anxiety, accounting for approximately 2% of the total effect, rather than a substantial explanatory mechanism. From a clinical perspective, this small magnitude indicates that physical activity alone is not a primary intervention for alleviating anxiety associated with severe family conflict. However, from a public health and preventive perspective, even indirect effects of limited magnitude can hold practical relevance in population-based research when the mediating factor is highly modifiable. Promoting physical activity remains a low-cost, accessible strategy that may offer marginal protective benefits for adolescents’ mental health. Nevertheless, the present findings provide only limited empirical support for the mediation claim, and the practical importance of physical activity as a buffer in this specific family dynamic should not be overstated.

This study found that gender had a small but statistically significant moderating effect on the association between parental marital conflict and adolescents’ physical activity. Specifically, under higher levels of parental marital conflict, male adolescents showed a somewhat stronger negative association with physical activity than female adolescents. This pattern may be tentatively understood through Gender Role Theory, which suggests that males and females are often socialized into different role expectations and coping tendencies in response to stress (McRae et al., 2008). Boys are generally encouraged to adopt more active and externalizing coping styles, whereas girls may be more likely to rely on internalizing or emotion-focused strategies. From this perspective, male adolescents may be somewhat more likely to use physical activity as a coping-related behavior when facing family stress, while female adolescents may be relatively less dependent on physical activity as a primary coping response (Williams and McGillicuddy-De Lisi, 1999). In high-conflict family contexts, lower support, fewer activity opportunities, and a tense home atmosphere may therefore be somewhat more strongly associated with lower levels of physical activity among boys. Previous research also suggests that females may show greater emotional sensitivity to social stressors (Whittle et al., 2017), although this does not necessarily translate into lower physical activity. By contrast, males may be somewhat more vulnerable to lower behavioral engagement when their preferred coping strategies are limited or unavailable (Goldfarb et al., 2019; She and Wang, 2025). However, given the modest magnitude of the moderation effect in the present study, these interpretations should remain cautious. Gender appears to function as only a limited source of individual difference in this pathway rather than as a strong explanatory mechanism. Accordingly, although gender may be relevant when considering adolescents’ behavioral responses to parental marital conflict, its theoretical importance in the present model should not be overstated, and other familial, emotional, and contextual factors are likely to play a larger role.

Taken together, this study adds to the literature on the association between parental marital conflict and adolescent anxiety by examining the statistical mediating role of physical activity and the modest moderating role of gender. Our findings suggest that higher parental marital conflict was associated with higher levels of adolescent anxiety and with lower levels of physical activity, which in turn was associated with higher anxiety. However, the indirect effect through physical activity was very small and should not be interpreted as evidence that physical activity is a strong protective mechanism against the psychological consequences of parental marital conflict. In addition, we identified a small but statistically significant moderating effect of gender in the marital conflict-physical activity pathway, with a stronger negative association among boys. From a practical perspective, promoting physical activity may be useful as one component of broader school- and community-based mental health support, but it should not be viewed as a standalone solution for adolescents exposed to high-conflict family environments.

This study has several limitations. First, all variables were assessed using self-reported measures, which may be affected by recall bias and social desirability bias. In particular, physical activity was measured using a self-reported questionnaire with modest internal consistency in the current sample. Although the Cronbach’s *α* coefficient of 0.608 may still be considered acceptable for a brief measure with fewer than six observed items, the relatively limited reliability of this measure may have introduced measurement error, especially in adolescents’ reports of activity intensity, duration, and frequency. The physical activity score was also right-skewed and contained high values, although sensitivity analyses using log-transformed and winsorized scores supported the robustness of the mediation result. Second, anxiety was measured using the GAD-2, which is a brief screening instrument rather than a diagnostic interview; therefore, the findings should be interpreted as reflecting anxiety symptom severity rather than clinical anxiety disorders. Third, because this study used a cross-sectional design, the results should be interpreted as associative rather than causal, and the temporal ordering among parental marital conflict, physical activity, and anxiety cannot be established. Fourth, the effect sizes observed in this study were relatively small, and the practical significance of the mediation and moderation effects should not be overstated. Fifth, the use of convenience sampling may limit the representativeness of the sample. Future studies should recruit more diverse participants and incorporate longitudinal or experimental designs, objective measures of physical activity, and a broader array of biopsychosocial variables such as academic stress, peer support, and psychological resilience.

## 5 Conclusion

This study examined the associations among parental marital conflict, adolescent anxiety, physical activity, and gender. Parental marital conflict was positively associated with adolescent anxiety, and physical activity showed a statistically significant but very small mediating effect in this association. Gender moderated the association between parental marital conflict and physical activity, with the negative association being more evident among boys. These findings suggest that physical activity may be a modest behavioral correlate within the broader link between family conflict and adolescent anxiety. Because the study was cross-sectional and the indirect effect was small, the findings should be interpreted cautiously and without causal overstatement.

## Data Availability

The datasets used and/or analyzed during the current study are not publicly available because they contain information from adolescent participants and are subject to privacy and ethical restrictions. The datasets are available from the corresponding author on reasonable request.
